# The evolving diagnosis and treatment paradigms of multiple myeloma in China: 15 years' experience of 1256 patients in a national medical center

**DOI:** 10.1002/cam4.5737

**Published:** 2023-02-21

**Authors:** Yang Yang, Jing Li, Wenjing Wang, Yawen Wang, Aziguli Maihemaiti, Liang Ren, Tianwei Lan, Chi Zhou, Panpan Li, Pu Wang, Xianmuseye Aihemaiti, Feifei Chen, Tianhong Xu, Jiadai Xu, Peng Liu

**Affiliations:** ^1^ Department of Hematology Zhongshan Hospital, Fudan University Shanghai China

**Keywords:** clinical observations, cytogenetics, multiple myeloma, prognosis

## Abstract

**Background:**

Significant advances in multiple myeloma (MM) over the past 15 years led to exciting changes in the management of MM patients in China, which in turn brought about the early diagnoses, precise risk stratifications, and improved prognoses.

**Methods:**

We summarized the dynamic changes in the management of newly diagnosed (ND) MM in a national medical center, crossing the old and novel drug era. Demographics, clinical characteristics, first‐line treatment, response rate, and survival were retrospectively collected among NDMMs diagnosed in Zhongshan Hospital Fudan University from January 2007 to October 2021.

**Results:**

Of the 1256 individuals, median age was 64 (range 31–89) with 45.1% patients >65 years. About 63.5% were male, 43.1% were at ISS stage III and 9.9% had light‐chain amyloidosis. Patients with abnormal ratio of free light chain (80.4%), extramedullary disease (EMD, 22.0%), and high‐risk cytogenetic abnormalities (HRCA, 26.8%) were detected by novel detection techniques. The best confirmed ORR was 86.5%, including 39.4% with CR. Short‐ and long‐term PFS and OS rates persistently increased each year along with increasing novel drug applications. Median PFS and OS were 30.9 and 64.7 months. Advanced ISS stage, HRCA, light‐chain amyloidosis and EMD independently predicted an inferior PFS. First‐line ASCT indicated a superior PFS. Advanced ISS stage, elevated serum LDH, HRCA, light‐chain amyloidosis, and receiving PI/IMiD‐based regimen versus PI+IMiD‐based regimen independently indicated a poorer OS.

**Conclusions:**

In brief, we illustrated a dynamic landscape of MM patients in a national medical center. Chinese MM patients evidently benefited from newly introduced techniques and drugs in this field.

## Graphical abstract

This is the first report illustrating the evolving patterns of multiple myeloma (MM) diagnosis and treatment and a reflection of the dynamic landscape of China's myeloma management. Chinese MM patients evidently benefited from comprehensive baseline evaluation and novel‐drug‐based (proteasome inhibitors [PIs] and immunomodulatory drugs [IMiDs]) standardized initial treatment.

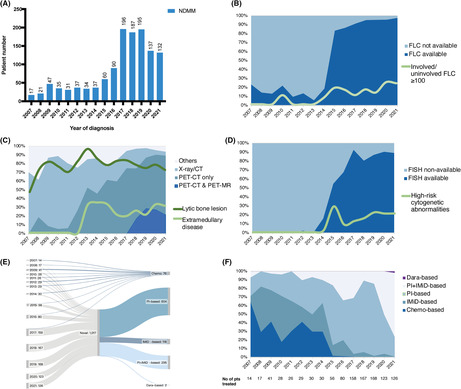

## 1 INTRODUCTION

Multiple myeloma (MM) is a malignancy of plasma cells derived from post‐germinal‐center B cells. Patients usually develop symptoms of end organ damages and have monoclonal protein (M‐protein) in serum and urine.^1^ The age‐standardized incidence rate (ASIR) of myeloma was 7.0 per 100,000 per year in the United States,^2^ which was composed of 1.8% of all new cancer cases. The incidence rate of myeloma in China was much lower (0.9/100,000) with an increasing trend.^3^, ^4^

The prognosis of MM has been greatly improved because of the introduction of novel drugs over the past decades.^5^ The 5‐year overall survival (OS) rate was 57.9% in the United States from 2012 to 2018 according to Surveillance, Epidemiology, and End Results Program (SEER) database and among different ethnic groups, the Asian population had the best prognosis.^2^ To the contrary, Chinese myeloma patients had an inferior prognosis, which was attributed to delayed diagnosis, accessibility to novel drugs, and lower rate of receiving autologous stem cell transplantation (ASCT). The 5‐year OS rate was lower than 50% in three famous myeloma centers, with only half of the patients receiving bortezomib‐based regimens.^3^ In another retrospective single‐center study, the 5‐year OS was 47.6%, while the proportion of patients receiving novel regimens was not referred to.^6^

Significant advances in diagnostic techniques and therapeutic regimens of MM over the past 15 years have led to exciting changes in the management of myeloma patients in China. Hematologists became more understood on this plasma cell disorder and in turn, more patients could be diagnosed at an early stage and undergo comprehensive and adequate examinations, including serum‐free light chain, fluorescence in situ hybridization (FISH), positron emission tomography/computed tomography (PET/CT), etc., which allowed precise risk stratifications. Treatment revolutions including novel drugs and maintenance therapy have made persistent disease remission possible.^7^, ^8^ Herein, we summarized the clinical characteristics and outcome of 1256 newly diagnosed MM (NDMM) patients in Zhongshan Hospital Fudan University over the past 15 years. We would like to illustrate the evolving management changes in myeloma in such a referral medical center in Shanghai and one of top five national medical centers in China. Our experience could work as a reflection of the dynamic landscape of China's myeloma centers.

## 2 MATERIALS AND METHODS

### 2.1 Patients

This study retrospectively enrolled NDMMs in Zhongshan Hospital Fudan University from January 2007 to October 2021 (Figure S1). The diagnosis of MM patients was in accordance with the International Myeloma Working Group (IMWG) diagnosis criteria.^9^ The study complied with the Declaration of Helsinki and was approved by the ethics committee (B2017‐031R). All data were collected and analyzed after the informed consents were signed by patients or their agents.

### 2.2 Clinical data

Data were collected from patients' electronical medical records, including demographics, Eastern Cooperative Oncology Group (ECOG) performance status (PS), clinical characteristics (including serum protein electrophoresis [SPEP], serum immunofixation electrophoresis [sIFE], International Staging System [ISS], bone marrow aspirate and biopsy, plasma cell FISH, serum lactate dehydrogenase [LDH], electrolytes, creatinine, hemoglobulin, lytic bone lesion, extramedullary disease [EMD], light‐chain amyloidosis [AL]), first‐line treatment regimens and duration, response rate, and survival. The definitions of double‐hit and triple‐hit myeloma were in accordance with the Mayo Clinic mSMART3.0.^10^ EMD was diagnosed by PET/CT scan, CT scan, or magnetic resonance imaging (MRI). The definitions of responses followed the IMWG consensus criteria for response in 2016.^11^

### 2.3 Statistical analysis

The Mann–Whitney and Wilcoxon rank sum tests were used to compare continuous variables. The Fisher's exact and $\chi^{2}$ tests were used to compare categorical variables. Progression‐free survival (PFS) was defined as the duration from diagnosis to disease progression or death, whichever comes first.^12^ Overall survival (OS) was defined as the duration from diagnosis to the last follow‐up date or death. Survival analyses were performed by the Kaplan–Meier method. Comparison among subgroups were performed via log‐rank test. The Cox proportional hazards models were used for univariate and multivariate analyses. Factors with *p*‐value ≤0.10 in univariate analyses were included in the multivariate model. All data were analyzed using SPSS 26.0 and R Studio 4.1.2.

## 3 RESULTS

### 3.1 Patient scale, clinical characteristics, and novel detection methods

**FIGURE 1:**
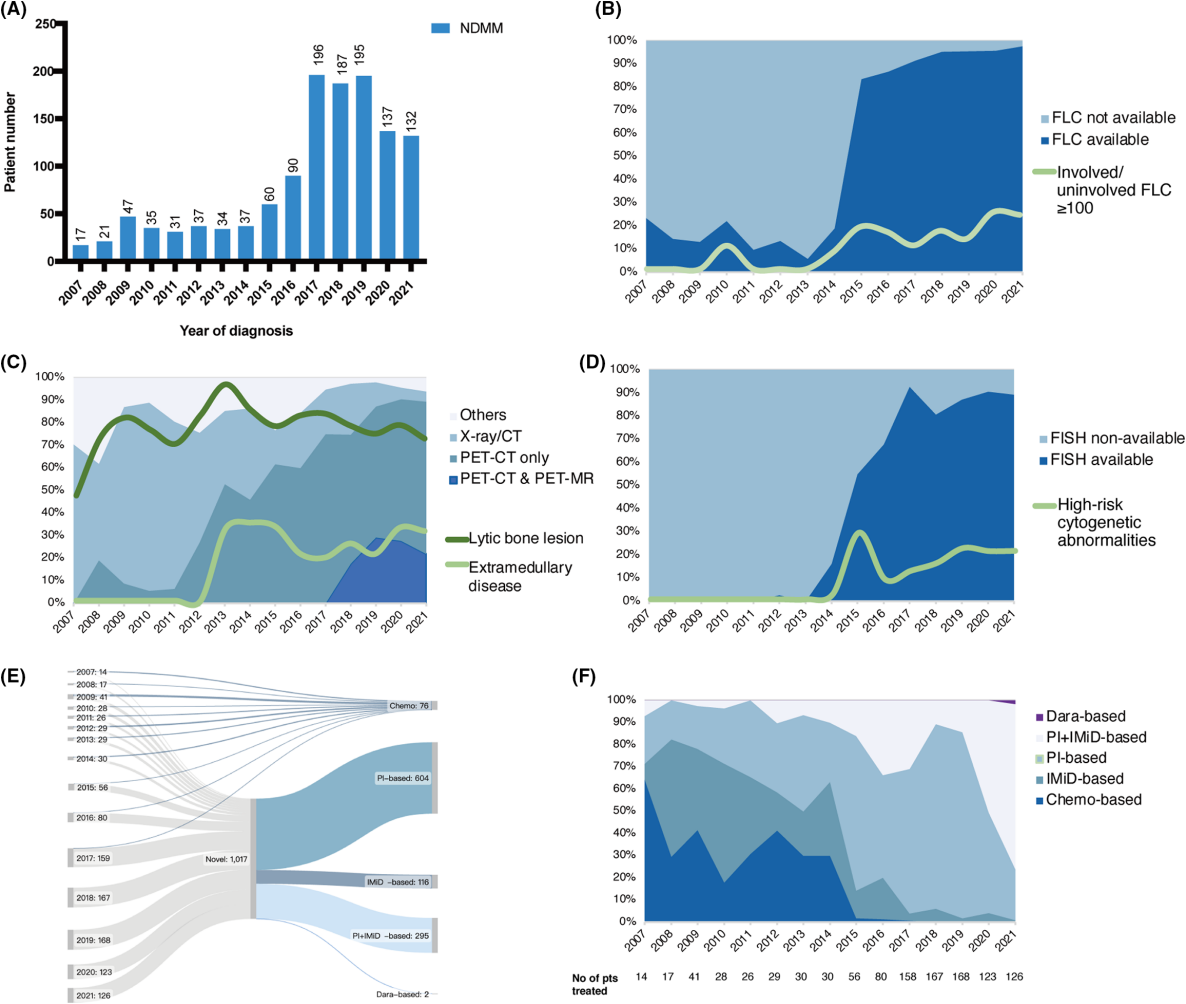
Expanding patient scale, advanced detection techniques, and novel regimens. (A) A growing number of MM patients were diagnosed in Zhongshan Hospital, Fudan University each year. (B) The rate of FLC started to increase after 2015. Since then, more patients with involved/uninvolved FLC ≥100 have been found out. (C) More patient received positron emission tomography/computed tomography (PET/CT) as bone evaluation instead of X‐ray/CT. At the same time, more patients were diagnosed with extramedullary disease because of the widespread utilization of PET/CT. (D) The fluorescence in situ hybridization (FISH) tests was routinely taken in NDMM after 2015, along with more patients with HRCA detected. (E) Sankey diagram of the number of patients who received chemotherapy/novel agents each year. (F) The proportion of patients who received chemotherapy/novel agents each year was shown in percentage stack area chart. A large proportion of patients started to receive regimens containing a single novel drug after 2015 and dual novel drugs after 2020.FLC, free light chain; CT, computed tomography; PET/CT, positron emission tomography/computed tomography; MR, magnetic resonance; FISH, fluorescence in situ hybridization; Dara, daratumumab; PI, proteasome inhibitor; IMiD, immunomodulatory drug; Chemo, chemotherapy.

The number of NDMM has increased from tens to nearly two hundred each year, contributed by early diagnosis and progressive understanding of MM. (Figure 1A). Patient clinical data were summarized in Table 1. The median age was 64 (range 31–89) with 566 (45.1%) >65 years. There were 798 (63.5%) male and 458 (36.5%) female patients, respectively. As for immunoglobulin subtypes, 621 (49.8%)/322 (25.8%)/179 (14.4%)/32 (2.6%)/9 (0.7%)/1 (0.1%) patients had IgG/IgA/light‐chain/IgD/IgM/IgE subtype. 20 (1.6%) had biclonal type and 62 (5.0%) had non‐secretory type. 10 patients only had positive sIFE records. 522 patients (43.1%) were at ISS stage III, and 244 (19.9%) had elevated serum LDH. An ECOG PS ≥2 was found in 450 (35.8%) patients. Light‐chain amyloidosis was observed at the myeloma diagnosis in 124 (9.9%) patients.

**TABLE 1:** Clinical characteristics of newly diagnosed multiple myeloma patients.

Total (*N* = 1256)		
Age, median (IQR)	64	(57–71)
>65 years old, *n* (%)	566	(45.1)
Gender, *n* (%)		
Male	798	(63.5)
Female	458	(36.5)
M‐protein type, *n* (%)^a^		
IgG	621	(49.8)
IgA	322	(25.8)
IgM	9	(0.7)
IgD	32	(2.6)
IgE	1	(0.1)
Light chain	179	(14.4)
Biclonal	20	(1.6)
Non‐secretory	62	(5.0)
Missing value	10	–
BM plasma cell%, median (IQR)	30	(10–50)
ISS stage, *n* (%)^a^		
I	312	(25.8)
II	376	(31.1)
III	522	(43.1)
Missing value	46	–
Elevated LDH, *n* (%)^a^	244	(19.9)
Missing value	29	–
Hypercalcemia, *n* (%)^a^	95	(7.7)
Missing value	16	–
Renal insufficiency, *n* (%)^a^	224	(18.0)
Missing value	13	–
Anemia, *n* (%)^a^	628	(50.2)
Missing value	5	–
Bone lesion, *n* (%)^a^	977	(78.8)
Missing value	16	–
Extramedullary disease, *n* (%)	276	(22.0)
ECOG, *n* (%)		
0	127	(10.1)
1	678	(54.0)
2	320	(25.5)
3	111	(8.8)
4	19	(1.5)
AL amyloidosis, *n* (%)	124	(9.9)
Cytogenetics abnormalities, *n* (%)^a^		
Del(17p)		
Positive	85	(10.1)
Negative	758	(89.9)
Missing value	413	–
Del(13q14)		
Positive	375	(45.1)
Negative	456	(54.9)
Missing value	425	–
Gain1q		
Positive	406	(51.9)
Negative	376	(48.1)
Missing value	474	–
t(4;14)		
Positive	124	(16.2)
Negative	643	(83.8)
Missing value	489	–
t(11;14)		
Positive	128	(16.8)
Negative	633	(83.2)
Missing value	495	–
t(14;16)		
Positive	18	(2.3)
Negative	748	(97.7)
Missing value	490	–
Double‐hit		
Positive	123	(16.4)
Negative	625	(83.6)
Missing value	508	–
Del(17p) plus gain1q	30	(4.0)
Del(17p) plus t(4;14)	2	(0.3)
Gain1q plus t(4;14)	79	(10.6)
Gain1q plus t(14;16)	12	(1.6)
Triple‐hit		
Positive	17	(2.3)
Negative	731	(97.7)
Missing value	508	–
Del(17p) plus gain1q plus t(4;14)	11	(1.5)
Gain1q plus t(4;14) plus t(14;16)	4	(0.5)
Del(17p) plus gain1q plus t(14;16)	1	(0.1)
Del(17p) plus t(4;14) plus t(14;16)	1	(0.1)

^a^ Percentages were calculated after removing missing values.

The disease landscape of myeloma patients in our center has changed over the years due to the application of several novel detection methods. Of the 960 patients who had sFLC results at baseline, 772 (80.4%) had an abnormal FLC ratio. About 190 (19.6%) had an involved/uninvolved FLC ≥100 and the positive rate increased by years, which was consistent with the growing detection rate (Figure 1B). More patients did PET/CT scans (from 0% in 2007 to 89.4% in 2021) as baseline bone evaluation instead of X‐ray or CT scans (from 70.6% in 2007 to 4.5% in 2021) (Figure 1C). This allowed a convenient detection of extramedullary disease (EMD) (276 patients, 22.0%) (Figure 1C, Table 1). The percentage of patients having high‐risk cytogenetic abnormalities (HRCA, including del17p, t(4;14), and/or t(14;16)) also increased by years, along with a growing number of patients receiving FISH test (from 0% in 2007 to 90.5% in 2021, Figure 1D). HRCA were detected in 207 patients (26.8%, among 772 patients with FISH results, Table 1). 123 and 17 were double‐ or triple‐hit myeloma. The most common HRCA combinations were shown in Table 1.

### 3.2 Dynamic changes in first‐line treatment regimens

A total of 604 (55.2%), 116 (10.6%), 295 (27.0%), and 76 (7.0%) patients received proteasome‐inhibitor‐ (PI‐)/immunomodulatory‐drug‐ (IMiD‐)/PI+IMiD−/chemo‐based first‐line treatment (Figure 1E). Two patients at advanced ISS stage received a quadlet regimen of daratumumab, bortezomib, lenalidomide, and dexamethasone (DVRd) as induction therapy. Overall, the ratio of different regimens these patients received as first‐line treatment has changed tremendously, as more patients would receive the regimen containing one novel agent after 2015. The increasing rate of receiving two‐novel‐agents‐containing regimen after 2020 was also in accordance with the policy that VRd triplet was covered by the national medical insurance that year (Figure 1F).

### 3.3 Treatment response

Of the 1093 patients who received initial treatment in Zhongshan Hospital, the best confirmed ORR (≥PR) was 86.5%, including 62.6% patients with ≥VGPR and 39.4% with CR (Table 2). The median cycle of first‐line treatment was seven (range 1–26). 121 (11.1%) patients received first‐line autologous stem cell transplantation (ASCT). Four hundred and five patients (56.0%, among 723 patients who finished induction therapy in Zhongshan Hospital) had maintenance therapies.

**TABLE 2:** Treatment outline in all patients.

	Total (*N* = 872)^a^	
Best confirmed response, *n* (%)		
ORR	754	(86.5)
VGPR or better	546	(62.6)
CR + sCR	344	(39.4)
VGPR	202	(23.2)
PR	208	(23.9)
MR	37	(4.2)
SD	75	(8.6)
Cycles, median (range)	7	(1–26)
First‐line ASCT, *n* (%)	121/1093	(11.1)
Maintenance, *n* (%)^b^	405/723	(56.0)
Lines of therapies received, median(range)	1	(1–9)

^a^ Only patients with evaluable response were included.

^b^ Patients who had progression disease, regimens changed or who were still in first‐line treatment were not calculated.

### 3.4 Improvement of patient survival

**FIGURE 2:**
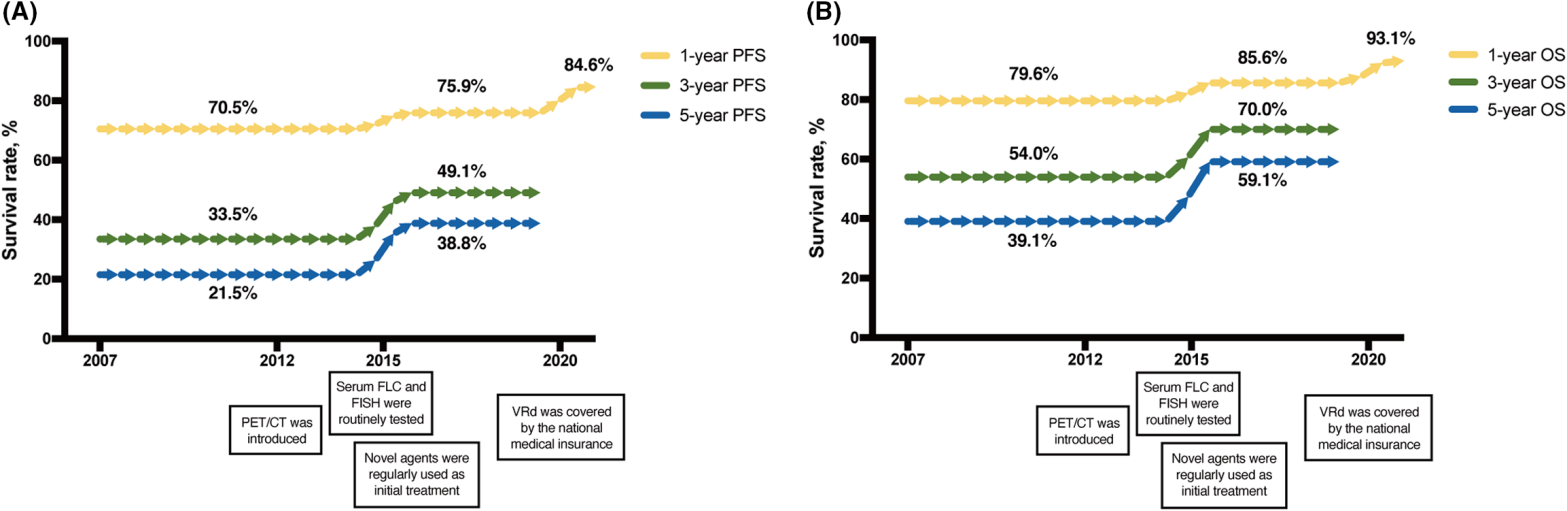
Improving patient survival during the 15 years. (A) The dynamic flow of PFS rate. An increase in the utilization of PET/CT started from 2012. Since 2015, sFLC and FISH has become routine tests for NDMM and the proportion of patients receiving one novel agent begun to arise. VRd has been covered by the national medical insurance since 2020, and more patients started to receive this regimen. The 1‐year PFS rates before 2015, from 2015 to 2019 and after 2020 were 70.5%, 75.9%, and 84.6%, respectively. The 3‐year PFS rates before 2015 and after 2015 were 33.5% and 49.1%, respectively. The 5‐year PFS rates before 2015 and after 2015 were 21.5% and 38.8%, respectively. The 3‐ and 5‐year PFS rates of patients diagnosed after 2020 were not available due to limited follow‐up time. (B) The dynamic flow of OS rate. The 1‐year OS rates before 2015, from 2015 to 2019 and after 2020 were 79.6%, 85.6%, and 93.1%, respectively. The 3‐year OS rates before 2015 and after 2015 were 54.0% and 70.0%, respectively. The 5‐year OS rates before 2015 and after 2015 were 39.1% and 59.1%, respectively. The 3‐ and 5‐year OS rates of patients diagnosed after 2020 were not available due to limited follow‐up time. PFS, progression‐free survival; PET/CT, positron emission tomography/computed tomography; sFLC, serum free light chain; FISH, fluorescence in situ hybridization; NDMM, newly diagnosed multiple myeloma; VRd, bortezomib lenalidomide and dexamethasone; OS overall survival.

We observed a persistent improvement of patient survival during these years, which was tightly correlated with the introduction of new diagnostic tools and the routine application of novel agents (Figure 2A,B). The 1‐year PFS rate before 2015 was 70.5% and increased to 75.9% after 2015, when novel detection methods such as sFLC and FISH tests were routinely taken at diagnosis. Novel agents were also regularly used as initial treatment. The same trend was seen in 3‐year PFS (33.5%–49.1%) and 5‐year PFS (21.5%–38.8%). A second rise of 1‐year PFS was seen in 2020, when VRd was covered by the national medical insurance and that it became more affordable to patients from 70.5% (2007–2014) to 84.6% (2020–2021). The improvement of OS rate was shown in Figure 2B (1‐year OS rate: 79.6%–85.6%–93.1%; 3‐year OS rate: 54.0%–70.0%; 5‐year OS rate: 39.1%–59.1%). Of note, the 3‐ and 5‐year survival rates were not available for patients diagnosed after 2020 due to limited follow‐up time.

**FIGURE 3:**
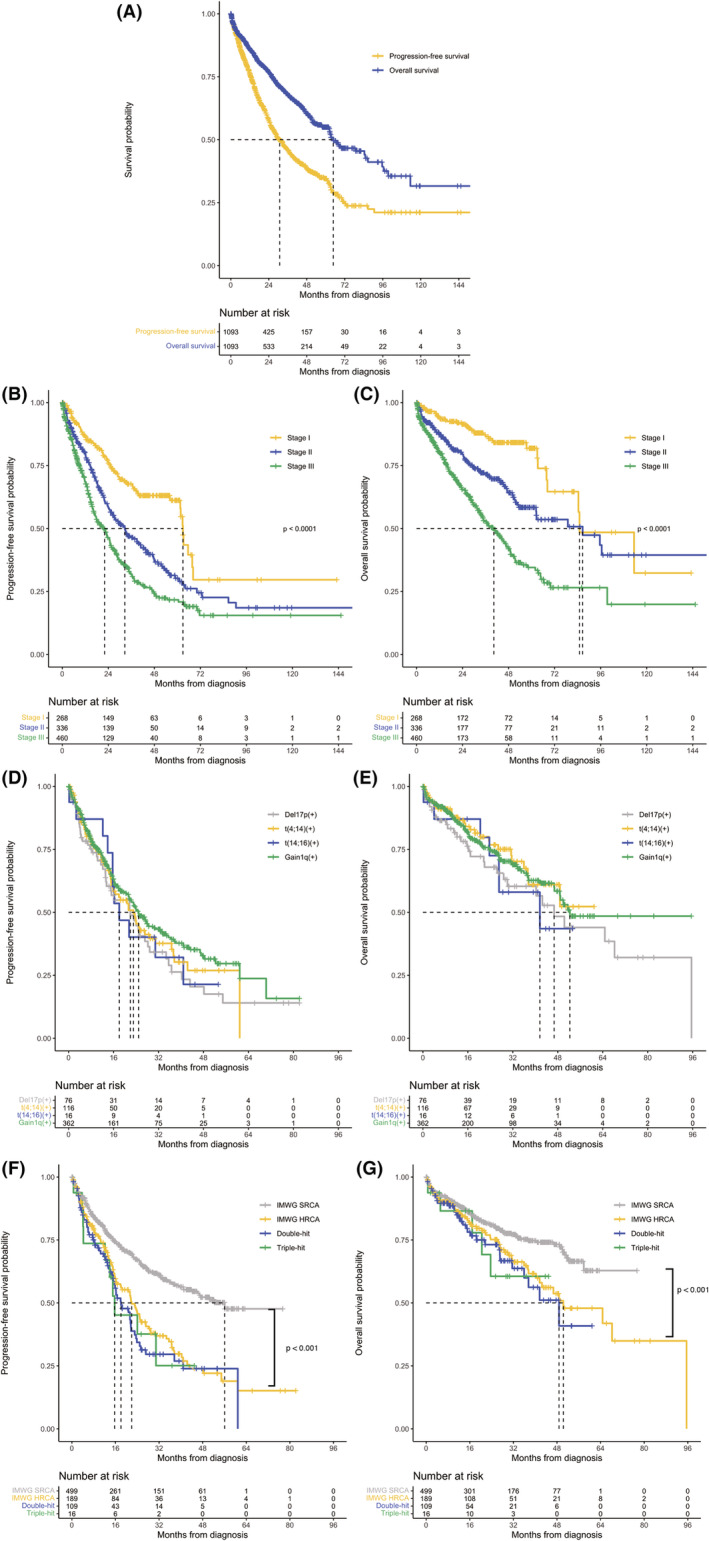
Patient survival outcomes. (A) PFS and OS of the whole cohort. (B) PFS, stratified by ISS stage. (C) OS, stratified by ISS stage. (D) PFS of patients with del17p, t(4;14), t(14;16), and gain1q. (E) OS of patients with del17p, t(4;14), t(14;16), and gain1q. (F) PFS of patients with SRCA, HRCA, double‐hit, and triple‐hit. (G) OS of patients with SRCA, HRCA, double‐hit, and triple‐hit. PFS, progression‐free survival; OS, overall survival; ISS, international staging system; SRCA, standard‐risk cytogenetic abnormalities; HRCA, high‐risk cytogenetic abnormalities.

With a median follow‐up time of 33.3 months, the median PFS (mPFS) (95% CI) of the whole cohort was 30.9 (27.0–34.7) months. Median OS (mOS) (95% CI) was 64.7 (52.2–77.3) months (Figure 3A). In ISS stage I–III groups, mPFS was 62.9, 32.6, and 21.5 months (*p* < 0.001, Figure 3B) and mOS was 84.9, 86.6, and 40.3 months (*p* < 0.001, Figure 3C).

For different cytogenetic abnormalities, patients with del17p/t(4;14)/t(14;16)/gain1q had mPFS of 21.9/23.1/18.0/24.9 months, respectively (Figure 3D). Patients with high‐risk cytogenetic abnormalities had shorter mPFS (21.8 months, standard‐risk cytogenetic abnormalities [SRCA]: 56.1 months, *p* < 0.001). Double‐hit and triple‐hit myeloma had mPFS of 18.5 and 15.7 months, respectively (Figure 3F). Patients with del17p /t(14;16)/gain1q had mOS of 46.7/41.6/52.3 months, while the mOS of t(4;14) (+) group was not reached (Figure 3E). HRCA patients also had a poorer mOS than SRCA (50.3 months vs. NR, *p* < 0.001) and double‐hit myeloma had a mOS of 46.7 months (Figure 3G). The mOS of triple‐hit myeloma was not reached, partially due to the limited patient number.

### 3.5 Prognostic factors

We wondered which were the factors that affected the prognosis of myeloma patients the most in a large‐scale cohort with a relatively long‐term follow‐up. In the univariate analysis of PFS (Table 3), male (*p* = 0.037), ISS stage II versus I (*p* < 0.001) /III versus I (*p* < 0.001), elevated serum LDH (*p* < 0.001), HRCA (*p* < 0.001), AL amyloidosis (*p* < 0.001), EMD (*p* = 0.003), PI/IMiD‐based initial treatment versus PI+IMiD‐based (*p* < 0.001), and chemo‐only versus PI+IMiD‐based (*p* < 0.001) were inferior factors. First‐line ASCT indicated a superior PFS (*p* < 0.001). For OS, ISS stage II versus I (*p* < 0.001) /III versus I (*p* < 0.001), elevated serum LDH (*p* < 0.001), HRCA (*p* = 0.011), AL amyloidosis (*p* < 0.001), PI/IMiD‐based initial treatment versus PI+IMiD‐based (*p* < 0.001), and chemo‐only versus PI+IMiD‐based (*p* < 0.001) indicated an inferior PFS while first‐line ASCT indicated a superior OS (*p* < 0.001).

**TABLE 3:** Univariate and multivariate cox regression analysis.

	Univariate analysis			Multivariate analysis		
**Variate**	HR	95% CI	*p*‐value	HR	95% CI	*p*‐value
PFS						
Age >65 vs. age ≤65	1.178	0.993–1.399	0.061	–	–	–
Male vs. female	0.825	0.688–0.988	0.037*	–	–	–
ISS stage						
Stage 2 vs. Stage 1	1.911	1.475–2.477	<0.001***	1.601	1.104–2.321	0.013*
Stage 3 vs. Stage 1	2.752	2.153–3.518	<0.001***	2.611	1.876–3.634	<0.001***
Elevated serum LDH	1.863	1.521–2.281	<0.001***	–	–	–
IMWG HRCA vs. SRCA	1.918	1.507–2.442	<0.001***	1.732	1.341–2.237	<0.001***
AL amyloidosis vs. pure MM	1.841	1.416–2.394	<0.001***	2.190	1.553–3.089	<0.001***
Extramedullary disease	1.347	1.105–1.642	0.003**	1.988	1.511–2.616	<0.001***
First‐line ASCT vs. no first‐line ASCT	0.324	0.225–0.466	<0.001***	0.386	0.231–0.644	<0.001***
Initial treatment regimen						
PI/IMiD‐based vs. PI+IMiD‐based	1.963	1.366–2.821	<0.001***	–	–	–
Chemo‐only vs. PI+IMiD‐based	1.642	1.303–2.069	<0.001***	–	–	–
OS						
Age > 65 vs. age < =65	1.212	0.976–1.505	0.082	–	–	–
Male vs. female	0.899	0.717–1.128	0.359	–	–	–
ISS Stage						
Stage 2 vs. Stage 1	2.195	1.514–3.182	<0.001***	2.084	1.184–3.668	0.011*
Stage 3 vs. Stage 1	4.093	2.895–5.786	<0.001***	3.828	2.308–6.347	<0.001***
Elevated serum LDH	2.782	2.199–3.520	<0.001***	1.866	1.302–2.675	0.001**
IMWG HRCA vs. SRCA	1.530	1.104–2.122	0.011	1.470	1.038–2.083	0.030
AL amyloidosis vs. pure MM	2.738	2.031–3.692	<0.001	2.711	1.811–4.057	<0.001
Extramedullary disease	1.061	0.815–1.380	0.660	–	–	–
First‐line ASCT vs. no first‐line ASCT	0.294	0.178–0.486	<0.001	–	–	–
Initial treatment regimen						
PI/IMiD‐based vs. PI+IMiD‐based	2.368	1.478–3.794	<0.001	1.697	1.113–2.587	0.014
Chemo‐only vs. PI+IMiD‐based	2.149	1.550–2.980	<0.001	–	–	–

*p < 0.05; **p < 0.01; ***p < 0.001.

In the multivariate analysis (Table 3), we confirmed advanced ISS stage (stage II vs. I, HR 1.601, *p* = 0.013; stage III vs. I, HR 2.611, *p* < 0.001), HRCA (HR 1.732, *p <* 0.001), light‐chain amyloidosis (HR 2.190, *p <* 001), and EMD (HR 1.988, *p <* 001) as factors of an inferior PFS. First‐line ASCT was a factor of a superior PFS (HR 0.386, *p* < 0.001). For OS, advanced ISS stage (stage II vs. I, HR 2.084, *p* = 0.011; stage III vs. I, HR 3.828, *p <* 001), elevated serum LDH level (HR 1.866, *p* = 0.001), HRCA (HR 1.470, *p* = 0.03), light‐chain amyloidosis (HR 2.711, *p <* 0.001), and receiving PI/IMiD‐based regimen versus PI+IMiD‐based regimen (HR 1.697, *p* = 0.014) indicated a poorer prognosis.

## 4 DISCUSSION

Herein, we illustrated the diagnosis and treatment landscape of 1256 MM patients in a national medical center in China over the past 15 years. This is, to our acknowledge, not only the largest scale of Chinese MM cohort study reported, but the first attempt to finely illustrate the impact of dynamic changes in diagnostic and therapeutic evolutions on MM patients as well.

The number of NDMM has risen sharply over the one and half decades. We started M‐protein screening in 2014. The screening was composed of sequentially SPEP screening, sIFE plus sFLC confirmation (if SPEP was positive) and further diagnostic examinations (if the previous lab results were positive). A summary of the screening was made by Li et al., that from 2014 to 2017, 151 of the 335 patients were identified through screening, who might otherwise be diagnosed much later because they just walked into the clinic for other health problems.^13^ An updated version of follow‐up has been summarized and is currently under review. Moreover, we also developed one of the earliest multi‐disciplinary team (MDT) of myeloma in China, which could potentially raise the awareness of non‐hematological specialists to identify myeloma‐defining symptoms. Therefore, patients could be referred to a hematologist properly.

Patients were relatively young with a median age of 64 years. The percentage of patients at advanced ISS stage was 43.1%, which was similar with those previously reported (29.7%–48.3%).^3^, ^6^, ^14^ The application of novel techniques enabled early diagnoses and delicate risk stratifications. As sFLC was routinely tested after 2015, more patients with abnormal rFLC were identified and those whose rFLC exceeded 100 (in accordance with the 2014 IWMG diagnosis criteria of “SLIM CRAB”) could be detected in time. More EMD cases occurred as the start of frequent applications of PET/CT scan. In total, 67.4% patients took PET/CT scan at diagnosis, 12.5% patients received PET/CT plus PET/MR scan for bone evaluation, and 22.0% patients had EMD at diagnosis. Among them, 219 had osseous EMD, 30 patients had soft tissue plasmacytoma, and 27 had both. The percentage of EMD was much higher than that reported by Verettoni et al. in 2009 (CT or MRI as evaluation tool) (7%) but similar with that in one recent single‐center study of Chinese patients (22.4%), where PET/CT scans were more frequently used.^6^, ^15^ The increased sensitivity of image detection methods led to a relatively high rate of EMD. Our team has recently summarized the PET/CT patterns of NDMM patients. Not only EMD but the PET pattern (diffuse/mixed patterns vs. normal or focal pattern) indicated a poorer prognosis, highlighting the importance of PET/CT.^16^ Moreover, we found an increasing number of HRCA patients, as we started to routinely made FISH tests as a daily management at diagnosis and at relapse after 2016.

The prognoses of myeloma patients were greatly improved by novel agents. In the era of chemotherapy, treatment options for MM were limited. Reported by Kyle et al., a cohort of 1027 NDMMs had a mOS of only 33 months, with no improvement from 1985 through 1998.^17^ The introduction of thalidomide plus dexamethasone deepened the response rate and prolonged the PFS of transplant‐ineligible NDMMs.^18^ In transplant‐eligible patients, results from HOVON‐65/GMMG‐HD4 and IFM2005‐01 supported a superior effect of novel‐agent‐containing regimens (bortezomib, doxorubicin, and dexamethasone [PAD] or bortezomib and dexamethasone [Vd]) over vincristine‐doxorubicin‐dexamethasone (VAD).^19^, ^20^ The updated analysis of SWOG S0777 strongly supported VRd triplet as an appropriate standard of care in NDMM because of the superior PFS and OS in the VRd arm.^21^

As shown in Figure 1E,F, before 2014, around 40% patients in Zhongshan Hospital each year received only chemotherapy such as VAD or melphalan‐prednisone (MP). Thalidomide was an option and bortezomib was rarely used. The new drug application did not greatly increase until 2015. In 2021, over 70% patients could receive a triplet regimen containing one PI and one IMiD (most frequently VRd). These changes were contributed by the improved accessibility of novel drugs and the expanding indications covered by the national medical insurance policy. Extreme improvement on patient prognoses was observed over the years, as the PFS and OS rates gradually increases, as shown in Figure 2A,B. This demonstrated not only the great potential of novel agents, but also the advanced detection techniques and complete baseline examinations on NDMM.

With 92.9% patients receiving at least one novel drug, our cohort had a mPFS of 30.9 months and a mOS of 64.7 months. The survival was apparently prolonged, compared with that in a cohort from three general hospitals and famous myeloma centers from 2008 to 2011 (mPFS: 26 months, mOS: 54 months), of which only 49.8% patients received bortezomib‐containing regimens.^3^ The 5‐year OS rate of our cohort was 55.0%, which was comparable with that reported from 2012 to 2018 by SEER database (57.9%). Furthermore, in subgroup analysis, we found a superior mPFS of 46.8 months in the PI+IMiD group (PI/IMiD group: 28.9 months, chemo‐only group: 20.5 months, *p* < 0.001, Figure S2A) while the mOS was not reached (PI/IMiD group: 62.4 months, chemo‐only group: 38.1 months, *p* < 0.001, Figure S2B). Benefit was further proved by the univariate and multivariate models. In brief, more patients can benefit from the improvement of new drug accessibility.

In our cohort, only 11.1% patients received a first‐line ASCT, which was much lower than the proportion in western countries. In a real‐world study in Australia and New Zealand, the utilization rate of first‐line ASCT was 67% and the overall utilization rate was 76%.^22^ Two recent phase 3 studies proved the significance of ASCT over new drugs on prolonging PFS significance in NDMM.^8^, ^23^ In our cohort, patients receiving ASCT had an extreme superior mPFS of 90.6 months and an mOS not reached than patients without first‐line ASCT (mPFS: 27.4 months, *p* < 0.0001; mOS: 62.4 months, *p* < 0.0001; Figure S3). In our regression model, we also confirmed a positive effect of ASCT on prolonged PFS. However, Chinese patients usually hesitate to receive transplantation, mainly because of their own perspective, financial capacity, and fear of side effects, and would rather choose non‐transplantation regimens, since it was of great convenience to take subcutaneous or even oral regimens in China's medical centers and clinics. The percentage of Chinese patients receiving first‐line ASCT was merely 12.6–14.4%.^3^, ^6^ The relatively low ASCT rate could potentially impede patients from taking the newest treatments in the future, because clinical trials of the next‐generation novel agents might only include patients who once received ASCT. Of note, we attempted to reflect the real situation in most hospitals in China, and this general issue needed to be paid close attention to and refined in the near future.

In this study, we also verified several risk factors for NDMM. Patients with HRCA had a poorer clinical outcome compared to those with SRCA. Double‐ and triple‐hit myeloma had a numeric shorter mPFS than HRCA group. In the multivariate analysis, we confirmed the inferior effects of advanced ISS stage, EMD, elevated serum LDH, HRCA, and light‐chain amyloidosis. Still, an unmet need was observed in the treatment of myeloma patients with high risk factors. Next‐generation novel agents such as daratumumab and carfilzomib could partially overcome the poor prognosis of MM with HRCA.^24^, ^25^ However, daratumumab was approved to treat transplant‐ineligible NDMM by the National Medical Products Administration (NMPA) in November 2021 (not covered by the national medical insurance). Carfilzomib was introduced to China in July 2021 with an approval of RRMM. We would anticipate the great benefits by more novel agents to Chinese MM patients in the near future. Furthermore, in a recently published study from our team, the effect of 1q gain/amplification was demonstrated. Patients with 1q gain/amplification had a worse PFS and OS than those without this CA, those who had 1q gain (copy number = 3) had similar survival outcome with those with 1q amplification (copy number >3). However, upfront ASCT could eliminate the adverse prognostic effect of 1q gain but not 1q amplification.^26^

Our study had several limitations. First, this was a retrospective study from a single center. Second, the missing values of FISH results could not be omitted. However, it has been only 6 years since our center started FISH tests in MM patients routinely and over two thirds of the missing values were from patient diagnosed before 2016. We believed the data could reflect the dynamic changes of MM patient management in our center. Therefore, after careful consideration, the FISH test results with missing values were listed, with the percentage of positive results calculated by excluding missing values. Third, we had only a few patients receiving ASCT, while only two patients received daratumumab‐based regimens (not covered by national medical insurance in NDMM) and the effect of this powerful agent could not be fully observed in our study. At last, the follow‐up time of our cohort was relatively short, partially because the number of NDMM began to increase only after 2014, and that the OS of patients receiving dual novel drugs was not mature. A longer follow‐up could be conducted to solve this problem.

## 5 CONCLUSIONS

In this study, we introduce the progressive improvement that a national medical center in China made to perfect its diagnostic and treatment paradigm of MM over the past 15 years. Newly introduced, sensitive techniques could provide accurate lab findings, discover rare disease types and lead to more precise risk stratification. Standardized therapies with the currently most accessible, newest regimens in China have brought extreme clinical benefits to NDMM patients.

## AUTHOR CONTRIBUTIONS

**Yang Yang:** Data curation (equal); formal analysis (lead); writing – original draft (lead). **Jing Li:** Data curation (equal); writing – review and editing (equal). **Wenjing Wang:** Data curation (equal). **Yawen Wang:** Data curation (equal). **Aiziguli Maihemaiti:** Data curation (equal). **Liang Ren:** Data curation (equal). **Tianwei Lan:** Data curation (equal). **Chi Zhou:** Data curation (equal). **Panpan Li:** Data curation (equal). **Pu Wang:** Data curation (equal). **Xianmuseye Aihemaiti:** Data curation (equal). **Feifei Chen:** Data curation (equal). **Tianhong Xu:** Data curation (equal). **Jiadai Xu:** Data curation (equal). **Peng Liu:** Conceptualization (lead); funding acquisition (lead); writing – review and editing (equal).

## CONFLICT OF INTEREST STATEMENT

The authors have no conflict of interests to declare.

## ETHICS APPROVAL STATEMENT

The study complied with the Declaration of Helsinki and was approved by the ethics committee (B2017‐031R). All data were collected and analyzed after the informed consents were signed by patients or their agents.

## Supporting information


Figure S1
Figure S2
Figure S3

## Data Availability

The dataset of myeloma patients in Zhongshan Hospital, Fudan University generated and analyzed during the current study was not publicly available due to the requirement of ethics committee of the hospital, in concerns of the protection of patients’ privacy.
