# Supplementary material for: The evolving diagnosis and treatment paradigms of multiple myeloma in China: 15 years' experience of 1256 patients in a national medical center
Source: Cancer Med. 2023 Feb 21;12(8):9604–14. doi: 10.1002/cam4.5737 (PMC10166961; doi:10.1002/cam4.5737)
Supplement: Supplementary file 1 — Figure S1 Figure S2 Figure S3 [file CAM4-12-9604-s001.pdf]

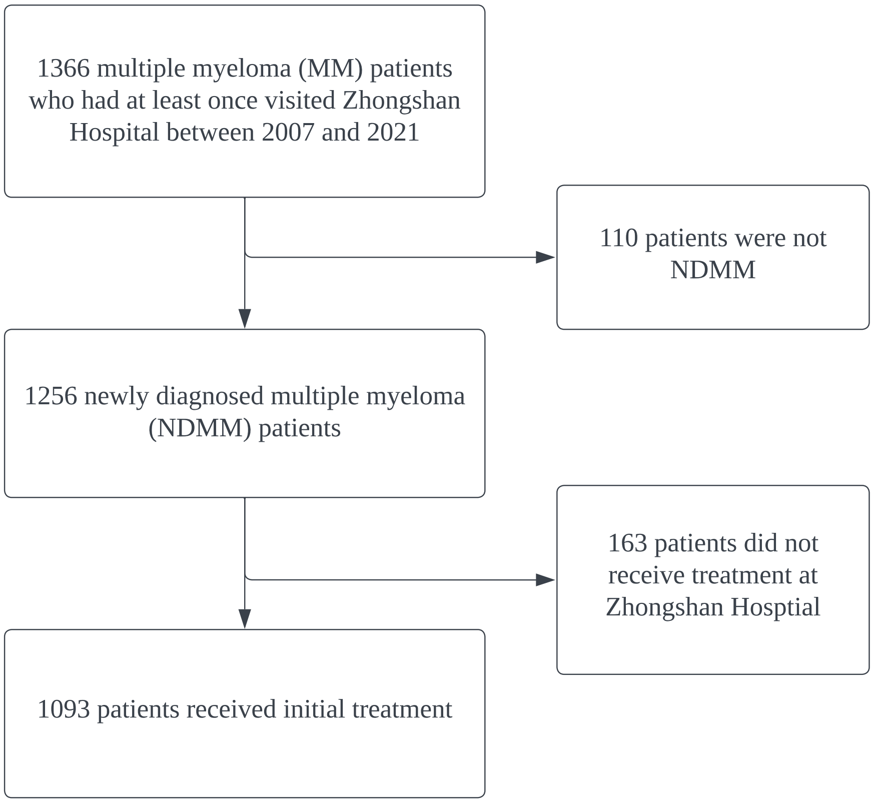

Supplementary Figure 1

A flowchart of newly diagnosed multiple myeloma (NDMM) patients enrolled in this study

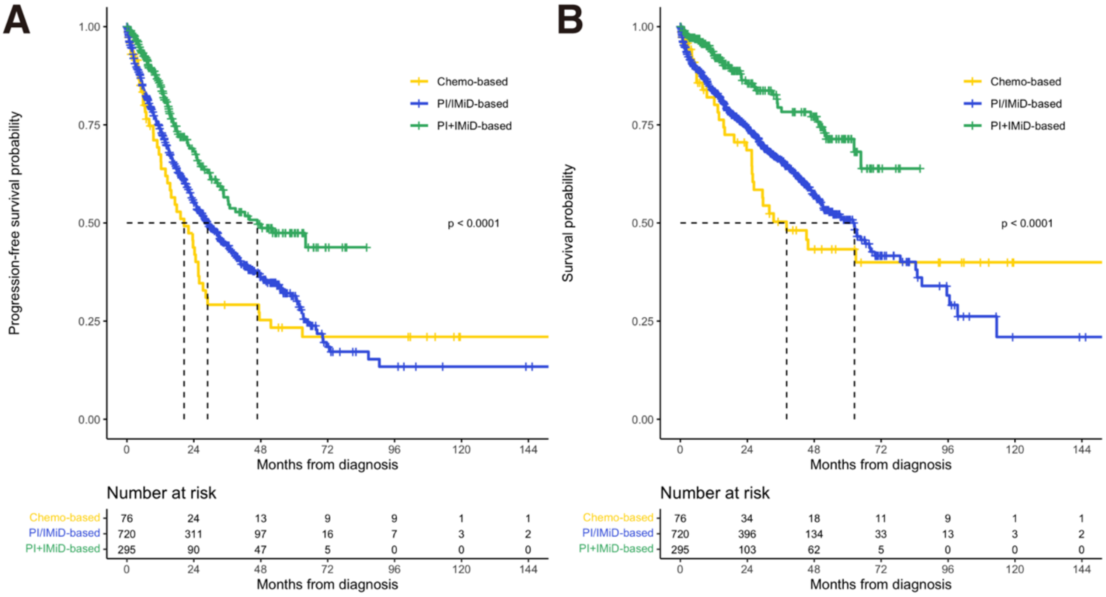

Supplementary figure 2

A. PFS of patients receiving chemotherapy, PI/IMiD-based or PI+IMiD-based induction therapy

B. OS of patients receiving chemotherapy, PI/IMiD-based or PI+IMiD-based induction therapy

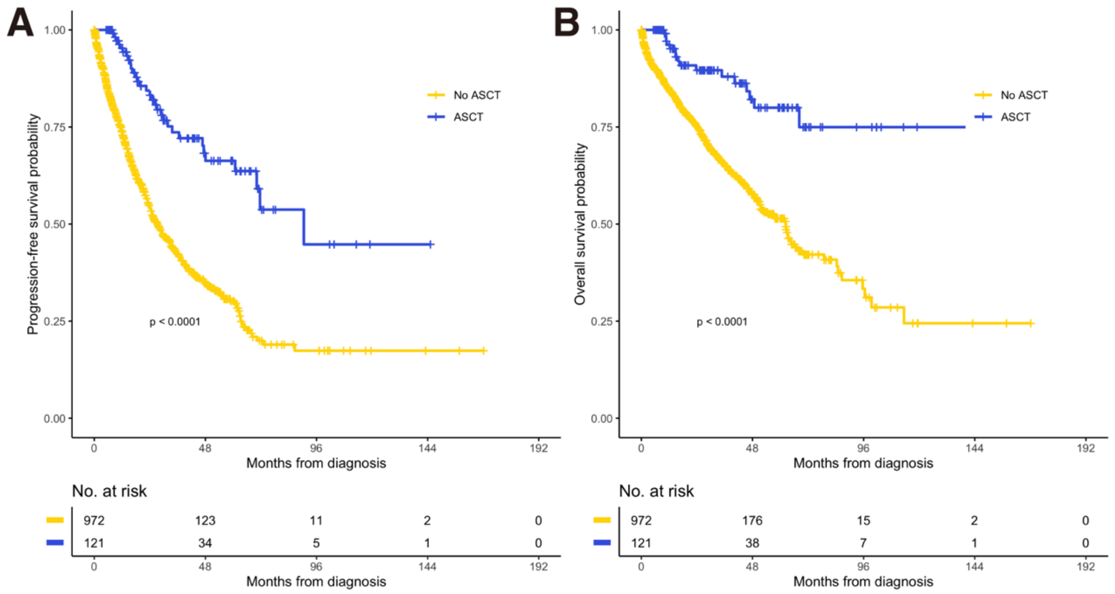

Supplementary figure 3

A. PFS of patients who had first-line ASCT and who did not have ASCT

B. OS of patients who had first-line ASCT and who did not have ASCT
